# Supplementary material for: VEGF-Related Germinal Polymorphisms May Identify a Subgroup of Breast Cancer Patients with Favorable Outcome under Bevacizumab-Based Therapy—A Message from COMET, a French Unicancer Multicentric Study
Source: Pharmaceuticals (Basel). 2020 Nov 23;13(11):414. doi: 10.3390/ph13110414 (PMC7700430; doi:10.3390/ph13110414)
Supplement: Supplementary file 1 [file pharmaceuticals-13-00414-s001.zip › Supplementary Files Manuscript Milano COMET Second Proof/Table S1.docx]

**Table S1:** Univariate analysis for the 13 SNPs according to PFS

|  |  |  | **No Progression** | **Progression** |  |  |  |
| --- | --- | --- | --- | --- | --- | --- | --- |
| **SNPs** | **Model** | **Genotype** | ***N* (%)** | ***N* (%)** | **HR** | **95% CI** | ***p*-value** |
| rs2010963 (*VEGFA*) |  |  |  |  |  |  |  |
|  | C-Allele Dominant | G/G | 3 (2.29%) | 128 (97.71%) | 1 | Referent |  |
|  |  | G/C or C/C | 16 (9.14%) | 159 (90.86%) | 0.77 | (0.61–0.97) | **0.026** |
| rs3025039 (*VEGFA*) |  |  |  |  |  |  |  |
|  | T-Allele Dominant | C/C | 15 (6.79%) | 206 (93.21%) | 1 | Referent |  |
|  |  | C/T or T/T | 4 (4.71%) | 81 (95.29%) | 0.91 | (0.71–1.20) | 0.495 |
| rs833061 (*VEGFA*) |  |  |  |  |  |  |  |
|  | T-Allele Dominant | T/T or T/C | 18 (7.44%) | 224 (92.56%) | 1 | Referent |  |
|  |  | C/C | 1 (1.56%) | 63 (98.44%) | 1.40 | (1.0–1.80) | **0.028** |
| rs699947 (*VEGFA*) |  |  |  |  |  |  |  |
|  | C-Allele Dominant | C/C or A/C | 18 (7.47%) | 223 (92.53%) | 1 | Referent |  |
|  |  | A/A | 1 (1.54%) | 64 (98.46%) | 1.30 | (1.0–1.80) | **0.042** |
| rs2229109 (*ABCB1*) |  |  |  |  |  |  |  |
|  | A-Allele Dominant | G/G | 16 (5.84%) | 258 (94.16%) | 1 | Referent |  |
|  |  | G/A or A/A | 3 (9.38%) | 29 (90.62%) | 1.10 | (0.73–1.60) | 0.741 |
| rs1045642 (*ABCB1*) |  |  |  |  |  |  |  |
|  | C-Allele Dominant | T/T | 7 (8.14%) | 79 (91.86%) | 1 | Referent |  |
|  |  | C/T or C/C | 12 (5.45%) | 208 (94.55%) | 1.20 | (0.90–1.50) | 0.243 |
| rs1128503 (*ABCB1*) |  |  |  |  |  |  |  |
|  | T-Allele Dominant | C/C | 6 (5.94%) | 95 (94.06%) | 1 | Referent |  |
|  |  | C/T or T/T | 13 (6.34%) | 192 (93.66%) | 0.96 | (0.75–1.20) | 0.762 |
| rs9582036 (*VEGFR1*) |  |  |  |  |  |  |  |
|  | C-Allele Dominant | A/A | 14 (8.97%) | 142 (91.03%) | 1 | Referent |  |
|  |  | C/A or C/C | 5 (3.33%) | 145 (96.67%) | 1.40 | (1.10–1.70) | **0.010** |
| rs2305948 (*VEGFR2*) |  |  |  |  |  |  |  |
|  | T-Allele Dominant | C/C | 14 (5.76%) | 229 (94.24%) | 1 | Referent |  |
|  |  | C/T or T/T | 5 (7.94%) | 58 (92.06%) | 1.00 | (0.78–1.40) | 0.756 |
| rs1870377 (*VEGFR2*) |  |  |  |  |  |  |  |
|  | A-Allele Dominant | T/A or A/A | 7 (5.51%) | 120 (94.49%) | 1 | Referent |  |
|  |  | T/T | 12 (6.7%) | 167 (93.3%) | 1.2 | (0.92–1.5) | 0.205 |
| rs2071559 (*VEGFR2*) |  |  |  |  |  |  |  |
|  | G-Allele Dominant | G/G or A/G | 13 (5.7%) | 215 (94.3%) | 1 | Referent |  |
|  |  | A/A | 6 (7.69%) | 72 (92.31%) | 0.91 | (0.70–1.20) | 0.495 |
| rs4073 (*IL8*) |  |  |  |  |  |  |  |
|  | T-Allele Dominant | T/T or T/A | 14 (5.93%) | 222 (94.07%) | 1 | Referent |  |
|  |  | A/A | 5 (7.14%) | 65 (92.86%) | 1.10 | (0.80–1.40) | 0.703 |
| rs11572080 (*CYP2C8*) |  |  |  |  |  |  |  |
|  | T-Allele Dominant | C/C | 13 (5.56%) | 221 (94.44%) | 1 | Referent |  |
|  |  | T/C or T/T | 6 (8.33%) | 66 (91.67%) | 0.96 | (0.73–1.30) | 0.750 |
