# Supplementary material for: VEGF-Related Germinal Polymorphisms May Identify a Subgroup of Breast Cancer Patients with Favorable Outcome under Bevacizumab-Based Therapy—A Message from COMET, a French Unicancer Multicentric Study
Source: Pharmaceuticals (Basel). 2020 Nov 23;13(11):414. doi: 10.3390/ph13110414 (PMC7700430; doi:10.3390/ph13110414)
Supplement: Supplementary file 1 [file pharmaceuticals-13-00414-s001.zip › Supplementary Files Manuscript Milano COMET Second Proof/Table S2.docx]

**Table S2:** Univariate analysis for clinicopathological features according to PFS

|  |  | **No Progression** | **Progression** |  |  |  |
| --- | --- | --- | --- | --- | --- | --- |
| **Patients and tumor characteristics** | **Modality** | ***N* (%)** | ***N* (%)** | **HR** | **95% CI** | ***p*-value** |
| Age |  |  |  |  |  |  |
|  | ≤ 55 | 11 (6.88%) | 149 (93.12%) | 1 | Referent |  |
|  | > 55 | 8 (5.48%) | 138 (94.52%) | 1.1 | (0.89–1.4) | 0.327 |
| Histology | Invasive ductal carcinoma  Invasive lobular carcinoma  Mixed (ductal & lobular)  Other | 15 (6.33%)  3 (7.89%)  0 (0%)  0 (0%) | 222 (93.67%)  35 (92.11%)  10 (100%)  13 (100%) | 1  0.64  0.68  0.03 | Referent  (0.64–1.3)  (0.47–1.7)  (1.1–3.3) | 0.641  0.688  0.031 |
| Menopausal status | Premenopausal  Postmenopausal | 7 (7.95%)  12 (5.63%) | 81 (92.05%)  201 (94.37%) | 1  0.98 | Referent  (0.76–1.3) | 0.908 |
| Performance status |  |  |  |  |  |  |
|  | 0 | 9 (5.23%) | 163 (94.77%) | 1 | Referent |  |
|  | 1-2 | 10 (7.46%) | 124 (92.54%) | 0.93 | (0.74–1.2) | 0.549 |
| Histological grade |  |  |  |  |  |  |
|  | I/II | 13 (8.23%) | 145 (91.77%) | 1 | Referent |  |
|  | III | 4 (3.31%) | 117 (96.69%) | 1.6 | (1.2–2.0) | **<0.001** |
| Tumor stage |  |  |  |  |  |  |
|  | pT0/pT1 | 10 (9.17%) | 99 (90.83%) | 1 | Referent |  |
|  | pT2 | 3 (3.57%) | 81 (96.43%) | 1.3 | (0.95–1.7) | 0.113 |
|  | pT3/pT4 | 2 (4.88%) | 39 (95.12%) | 1.2 | (0.79–1.7) | 0.459 |
| Axillary lymph node status |  |  |  |  |  |  |
|  | pN0 | 7 (8.43%) | 76 (91.57%) | 1 | Referent |  |
|  | pN+ | 8 (5.16%) | 147 (94.84%) | 1.1 | (0.8–1.4) | 0.721 |
| Subtype |  |  |  |  |  |  |
|  | Hormonal receptor positive | 15 (7.28%) | 191 (92.72%) | 1 | Referent |  |
|  | Triple-negative | 2 (3.12%) | 62 (96.88%) | 1.9 | (1.4–2.5) | **<0.001** |
| Prior (neo) adjuvant chemotherapy |  |  |  |  |  |  |
|  | No | 8 (8.33%) | 88 (91.67%) | 1 | Referent |  |
|  | Yes | 11 (5.24%) | 199 (94.76%) | 1.1 | (0.83–1.4) | 0.632 |
| Number of metastatic sites |  |  |  |  |  |  |
|  | < 3 | 14 (6.64%) | 197 (93.36%) | 1 | Referent |  |
|  | ≥ 3 | 0 (0%) | 24 (100%) | 1.3 | (0.85–2) | 0.230 |
| Metastatic-free survival |  |  |  |  |  |  |
|  | 0 month | 1 (5.88%) | 16 (94.12%) | 1 | Referent |  |
|  | ]0 - 24] months | 2 (2.82%) | 69 (97.18%) | 0.96 | (0.56–1.7) | 0.885 |
|  | > 24 months | 11 (7.48%) | 136 (92.52%) | 0.69 | (0.41–1.2) | 0.168 |
