# Supplementary material for: VEGF-Related Germinal Polymorphisms May Identify a Subgroup of Breast Cancer Patients with Favorable Outcome under Bevacizumab-Based Therapy—A Message from COMET, a French Unicancer Multicentric Study
Source: Pharmaceuticals (Basel). 2020 Nov 23;13(11):414. doi: 10.3390/ph13110414 (PMC7700430; doi:10.3390/ph13110414)
Supplement: Supplementary file 1 [file pharmaceuticals-13-00414-s001.zip › Supplementary Files Manuscript Milano COMET Second Proof/Table S3.docx]

**Table S3:** Univariate analysis for the 13 SNPs according to OS

|  |  |  | **Alive** | **Dead** |  |  |  |
| --- | --- | --- | --- | --- | --- | --- | --- |
| **SNPs** | **Model** | **Genotype** | ***N* (%)** | ***N* (%)** | **HR** | **95% CI** | ***p*-value** |
| rs2010963 (*VEGFA*) |  |  |  |  |  |  |  |
|  | C-Allele Dominant | G/G | 40 (30.53%) | 91 (69.47%) | 1 | Referent |  |
|  |  | G/C or C/C | 57 (32.57%) | 118 (67.43%) | 0.96 | (0.73–1.3) | 0.795 |
| rs3025039 (*VEGFA*) |  |  |  |  |  |  |  |
|  | T-Allele Dominant | C/C | 69 (31.22%) | 152 (68.78%) | 1 | Referent |  |
|  |  | C/T or T/T | 28 (32.94%) | 57 (67.06%) | 0.84 | (0.62–1.1) | 0.263 |
| rs833061 (*VEGFA*) |  |  |  |  |  |  |  |
|  | T-Allele Dominant | T/T or T/C | 85 (35.12%) | 157 (64.88%) | 1 | Referent |  |
|  |  | C/C | 12 (18.75%) | 52 (81.25%) | 1.6 | (1.1–2.1) | **0.005** |
| rs699947 (*VEGFA*) |  |  |  |  |  |  |  |
|  | C-Allele Dominant | C/C or A/C | 84 (34.85%) | 157(65.15%) | 1 | Referent |  |
|  |  | A/A | 13 (20%) | 52(80%) | 1.5 | (1.1–2.1) | **0.007** |
| rs2229109 (*ABCB1*) |  |  |  |  |  |  |  |
|  | A-Allele Dominant | G/G | 88 (31.12%) | 186 (67.88%) | 1 | Referent |  |
|  |  | G/A or A/A | 9 (28.12%) | 23 (71.88%) | 1.1 | (0.7–1.7) | 0.726 |
| rs1045642 (*ABCB1*) |  |  |  |  |  |  |  |
|  | C-Allele Dominant | T/T | 22 (25.58%) | 64 (74.42%) | 1 | Referent |  |
|  |  | C/T or C/C | 7 5(34.09%) | 145 (65.91%) | 0.88 | (0.66–1.2) | 0.413 |
| rs1128503 (*ABCB1*) |  |  |  |  |  |  |  |
|  | T-Allele Dominant | C/C | 34 (33.66%) | 67 (66.34%) | 1 | Referent |  |
|  |  | C/T or T/T | 63 (30.73%) | 142 (69.27%) | 1 | (0.76–1.4) | 0.879 |
| rs9582036 (*VEGFR1*) |  |  |  |  |  |  |  |
|  | C-Allele Dominant | A/A | 60 (38.46%) | 96 (61.54%) | 1 | Referent |  |
|  |  | C/A or C/C | 37 (24.67%) | 113 (75.33%) | 1.4 | (1.1–1.9) | **0.010** |
| rs2305948 (*VEGFR2*) |  |  |  |  |  |  |  |
|  | T-Allele Dominant | C/C | 72 (29.63%) | 171 (70.37%) | 1 | Referent |  |
|  |  | C/T or T/T | 25 (39.68%) | 38 (60.32%) | 0.76 | (0.54–1.1) | 0.129 |
| rs1870377 (*VEGFR2*) |  |  |  |  |  |  |  |
|  | A-Allele Dominant | T/A or A/A | 46 (36.22%) | 81 (63.78%) | 1 | Referent |  |
|  |  | T/T | 51 (28.49%) | 128 (71.51%) | 1.4 | (1–1.8) | **0.021** |
| rs2071559 (*VEGFR2*) |  |  |  |  |  |  |  |
|  | G-Allele Dominant | G/G or A/G | 73 (32.02%) | 155 (67.98%) | 1 | Referent |  |
|  |  | A/A | 24 (30.77%) | 54 (69.23%) | 1.1 | (0.78–1.4) | 0.721 |
| rs4073 (*IL8*) |  |  |  |  |  |  |  |
|  | T-Allele Dominant | T/T or T/A | 74 (31.36%) | 162 (68.64%) | 1 | Referent |  |
|  |  | A/A | 23 (32.86%) | 47 (67.14%) | 1 | (0.75–1.4) | 0.850 |
| rs11572080 (*CYP2C8*) |  |  |  |  |  |  |  |
|  | T-Allele Dominant | C/C | 72 (30.77%) | 162 (69.23%) | 1 | Referent |  |
|  |  | T/C or T/T | 25 (34.72%) | 47 (65.28%) | 0.85 | (0.61–1.2) | 0.318 |
