# Supplementary material for: VEGF-Related Germinal Polymorphisms May Identify a Subgroup of Breast Cancer Patients with Favorable Outcome under Bevacizumab-Based Therapy—A Message from COMET, a French Unicancer Multicentric Study
Source: Pharmaceuticals (Basel). 2020 Nov 23;13(11):414. doi: 10.3390/ph13110414 (PMC7700430; doi:10.3390/ph13110414)
Supplement: Supplementary file 1 [file pharmaceuticals-13-00414-s001.zip › Supplementary Files Manuscript Milano COMET Second Proof/Table S4.docx]

**Table S4:** Univariate analysis for clinicopathological features according to OS

|  |  | **Alive** | **Dead** |  |  |  |
| --- | --- | --- | --- | --- | --- | --- |
| **Patients and tumor characteristics** | **Modality** | ***N* (%)** | ***N* (%)** | **HR** | **95% CI** | ***p*-value** |
| Age |  |  |  |  |  |  |
|  | ≤ 55 | 53 (33.12%) | 107 (66.88%) | 1 | Referent |  |
|  | > 55 | 44 (30.14%) | 102 (69.86%) | 1 | (0.79–1.4) | 0.766 |
| Histology | Invasive ductal carcinoma  Invasive lobular carcinoma  Mixed (ductal & lobular)  Other | 74 (31.22%)  12 (31.58%)  2 (20%)  3 (23.08%) | 163 (68.78%)  26 (68.42%)  8 (80%)  10 (76.92%) | 1  1.1  0.95  1.1 | Referent  (0.7–1.6)  (0.47–1.9)  (0.55–2) | 0.805  0.880  0.873 |
| Menopausal status | Premenopausal  Postmenopausal | 26 (29.55%)  69 (32.39%) | 62 (70.45%)  144 (67.61%) | 1  0.95 | Referent  (0.7–1.3) | 0.731 |
| Performance status |  |  |  |  |  |  |
|  | 0 | 58 (33.72%) | 114 (66.28%) | 1 | Referent |  |
|  | 1-2 | 39 (29.10%) | 95 (70.90%) | 1.2 | (0.91–1.6) | 0.208 |
| Histological grade |  |  |  |  |  |  |
|  | I/II | 56 (35.44%) | 102 (64.56%) | 1 | Referent |  |
|  | III | 29 (23.97%) | 92 (76.03%) | 1.4 | (1.1–1.9) | **0.011** |
| Tumor stage |  |  |  |  |  |  |
|  | pT0/pT1 | 29 (35.78%) | 70 (64.22%) | 1 | Referent |  |
|  | pT2 | 20 (23.81%) | 64 (76.19%) | 1.4 | (0.98–1.9) | 0.065 |
|  | pT3/pT4 | 12 (29.27%) | 29 (70.73%) | 1.1 | (0.72–1.7) | 0.633 |
| Axillary lymph node status |  |  |  |  |  |  |
|  | pN0 | 25 (30.12%) | 58 (69.88%) | 1 | Referent |  |
|  | pN+ | 45 (29.03%) | 110 (70.97%) | 0.93 | (0.67–1.3) | 0.636 |
| Subtype |  |  |  |  |  |  |
|  | Hormonal receptor positive | 75 (36.41%) | 131 (63.59%) | 1 | Referent |  |
|  | Triple-negative | 9 (14.06%) | 55 (85.84%) | 2.4 | (1.7–3.3) | **< 0.001** |
| Prior (neo) adjuvant chemotherapy |  |  |  |  |  |  |
|  | No | 35 (36.46%) | 61 (63.54%) | 1 | Referent |  |
|  | Yes | 62 (29.52%) | 148 (70.48%) | 1 | (0.76–1.4) | 0.859 |
| Number of metastatic sites |  |  |  |  |  |  |
|  | < 3 | 60 (28.44%) | 151 (71.56%) | 1 | Referent |  |
|  | ≥ 3 | 6 (25%) | 18 (75%) | 1.2 | (0.72–1.9) | 0.525 |
| Metastatic-free survival |  |  |  |  |  |  |
|  | 0 month | 7 (41.18%) | 10 (58.82%) | 1 | Referent |  |
|  | ]0 - 24] months | 13 (18.31%) | 58 (81.69%) | 1.4 | (0.72–2.8) | 0.316 |
|  | > 24 months | 46 (31.29%) | 101 (68.71%) | 0.82 | (0.43–1.6) | 0.542 |
