# Supplementary material for: VEGF-Related Germinal Polymorphisms May Identify a Subgroup of Breast Cancer Patients with Favorable Outcome under Bevacizumab-Based Therapy—A Message from COMET, a French Unicancer Multicentric Study
Source: Pharmaceuticals (Basel). 2020 Nov 23;13(11):414. doi: 10.3390/ph13110414 (PMC7700430; doi:10.3390/ph13110414)
Supplement: Supplementary file 1 [file pharmaceuticals-13-00414-s001.zip › Supplementary Files Manuscript Milano COMET Second Proof/Table S5.docx]

**Table S5**: Summary of genotyping results by MassARRAY (AGENA) of 306 patients.

| **Gene SNPs** | **VEGFA** | | | | **VEGFR1** | **VEGFR2** | | | **IL8** | **CYP2C8** | **ABCB1** | | |
| --- | --- | --- | --- | --- | --- | --- | --- | --- | --- | --- | --- | --- | --- |
|  | **rs699947** | **rs833061** | **rs2010963** | **rs3025039** | **rs9582036** | **rs2071559** | **rs2305948** | **rs1870377** | **rs4073** | **rs11572080** | **rs2229109** | **rs1128503** | **rs1045642** |
| **Population** | C/C (97) | T/T (90) | G/G (131) | C/C (221) | A/A (156) | G/G (81) | C/C (243) | T/T (179) | T/T (92) | C/C (234) | G/G (274) | C/C (101) | T/T (86) |
|  | A/C (144) | T/C (152) | G/C (134) | C/T (80) | C/A (129) | A/G (147) | C/T (58) | T/A (113) | T/A (144) | T/C (68) | G/A (30) | C/T (146) | C/T (137) |
|  | A/A (65) | C/C (64) | C/C (41) | T/T (5) | C/C (21) | A/A (78) | T/T (5) | A/A (14) | A/A (70) | T/T (4) | A/A (2) | T/T (59) | C/C (83) |
| **Ancestral allele** | A | C | C | C | C | A | C | T | A | C | C | A | A |
| **Minor allele frequency** | 0.447 | 0.456 | 0.352 | 0.147 | 0.279 | 0.495 | 0.111 | 0.230 | 0.464 | 0.124 | 0.055 | 0.431 | 0.495 |
| **SNPs Functional Impact** | promoter | promoter | 5'-UTR | 3'-UTR | intronic | promoter | missense | missense | intronic | missense | missense | synonymous | synonymous |
