# Supplementary figures and images for: VEGF-Related Germinal Polymorphisms May Identify a Subgroup of Breast Cancer Patients with Favorable Outcome under Bevacizumab-Based Therapy—A Message from COMET, a French Unicancer Multicentric Study
Source: Pharmaceuticals (Basel). 2020 Nov 23;13(11):414. doi: 10.3390/ph13110414 (PMC7700430; doi:10.3390/ph13110414)

**Figure S1 :** Effect of significant clinical characteristics and polymorphisms according PFS

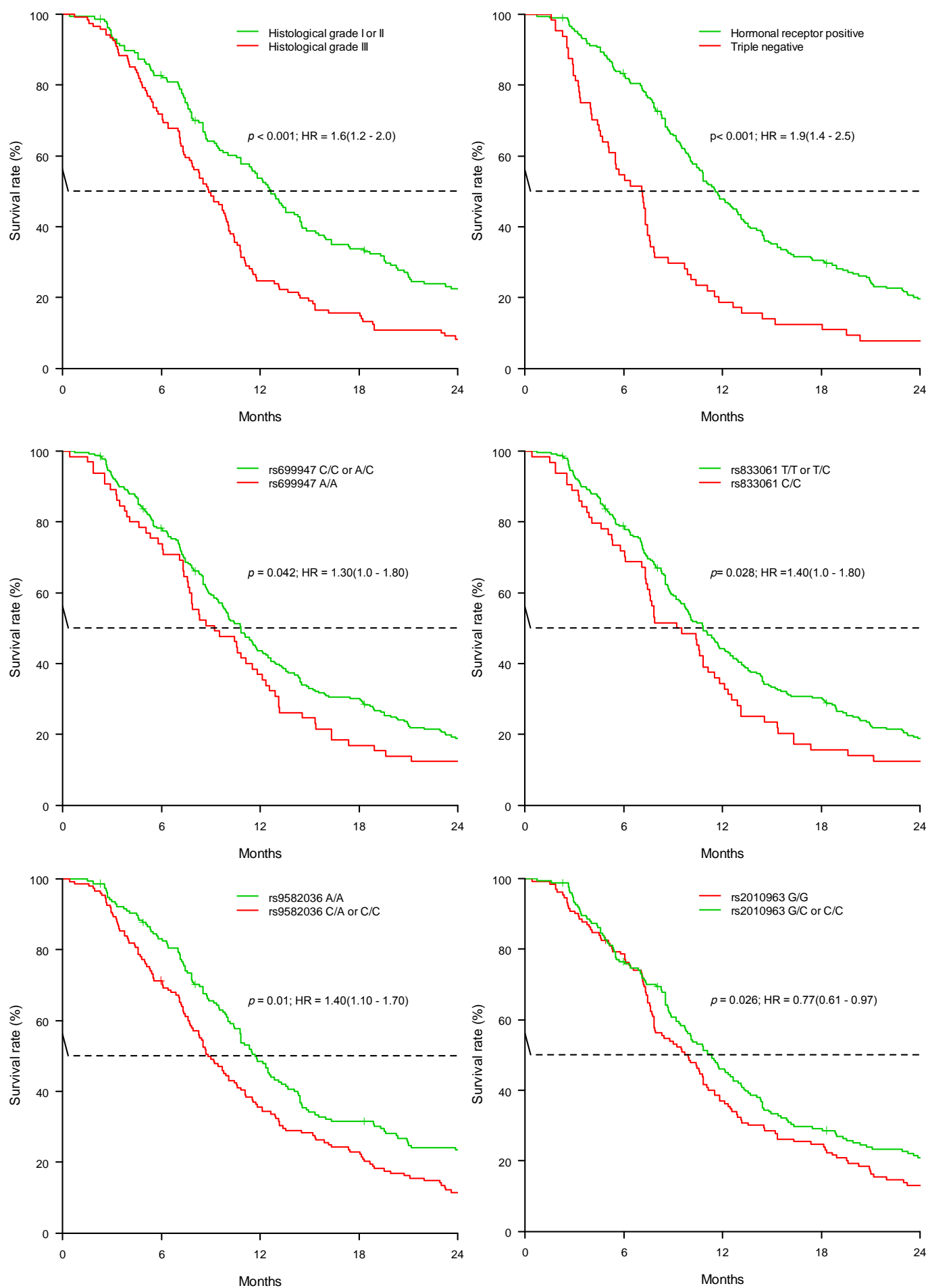

Supplement: Supplementary file 1 [file pharmaceuticals-13-00414-s001.zip › Supplementary Files Manuscript Milano COMET Second Proof/Figure S1.pdf]

**Figure S2 :** Effect of subtype and histological grade combinaison according to PFS

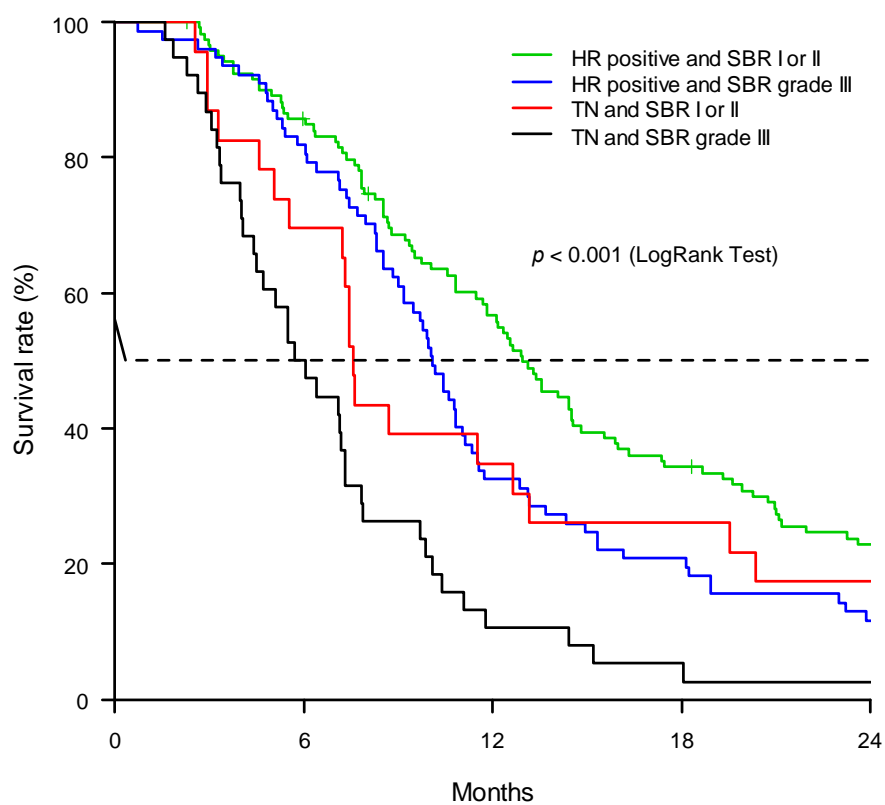

Supplement: Supplementary file 1 [file pharmaceuticals-13-00414-s001.zip › Supplementary Files Manuscript Milano COMET Second Proof/Figure S2.pdf]

**Figure S3 :** Effect of significant clinical characteristics and polymorphisms according OS

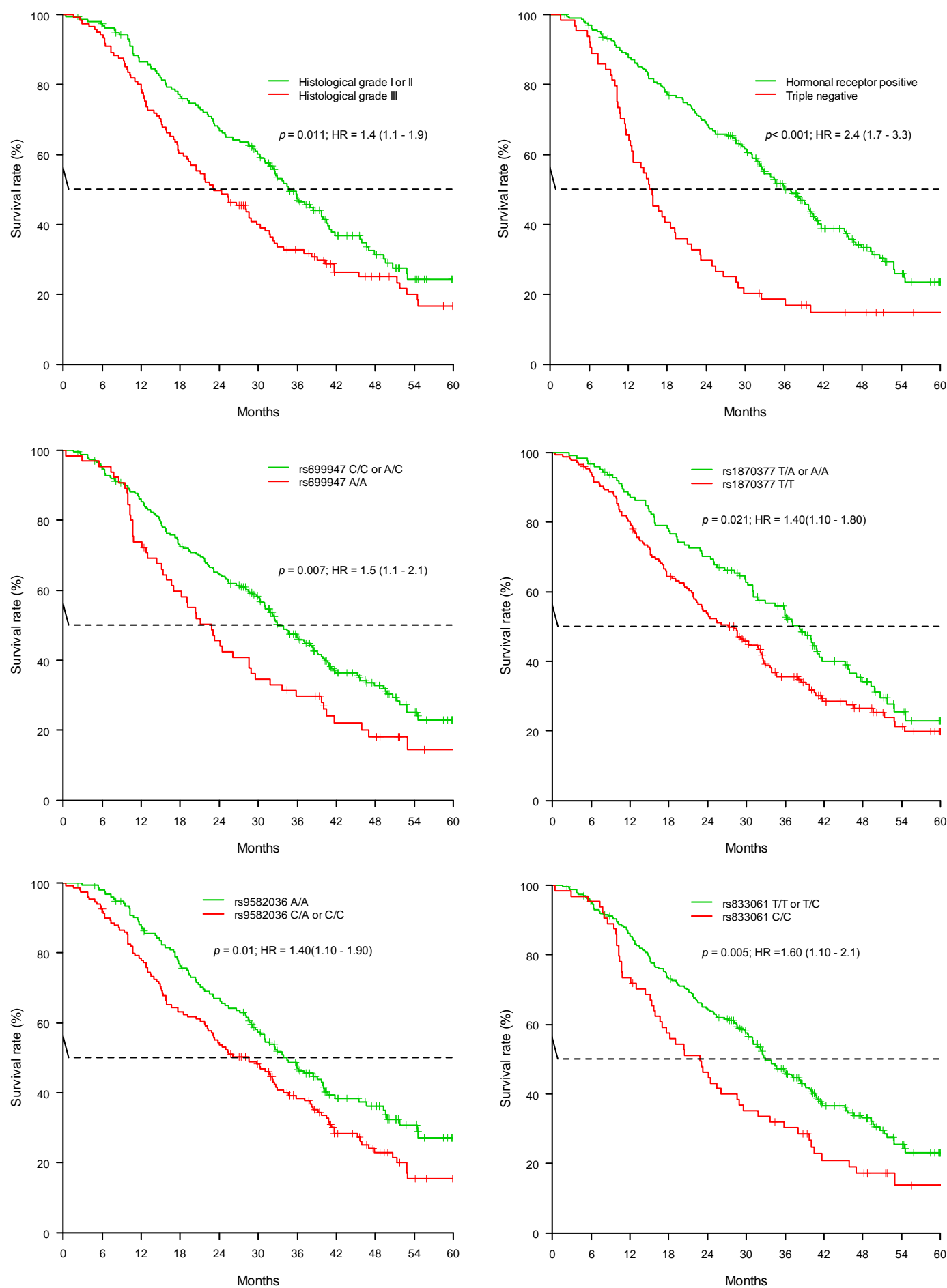

Supplement: Supplementary file 1 [file pharmaceuticals-13-00414-s001.zip › Supplementary Files Manuscript Milano COMET Second Proof/Figure S3.pdf]

**Figure S4 :** Effect of subtype and histological grade combinaison according to OS

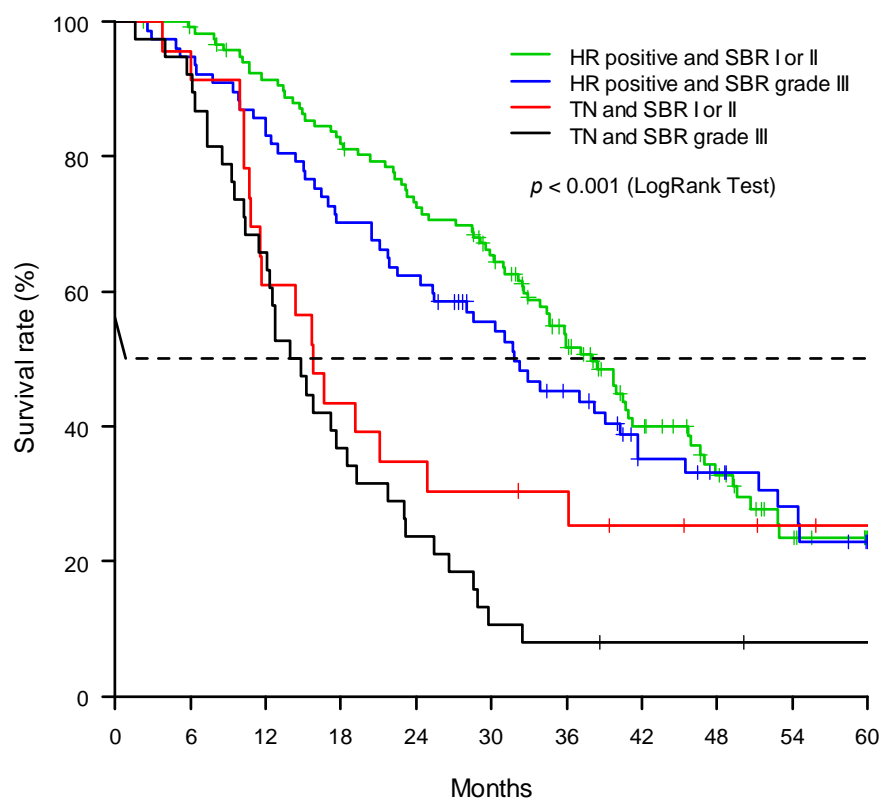

Supplement: Supplementary file 1 [file pharmaceuticals-13-00414-s001.zip › Supplementary Files Manuscript Milano COMET Second Proof/Figure S4.pdf]
